# Prognostic utility and clinical impact of adenosine stress perfusion CMR scan in patients with known coronary disease

**DOI:** 10.1186/1532-429X-13-S1-P118

**Published:** 2011-02-02

**Authors:** Vijay Dhakshinamurthy, Foord Rachel, Redha Boubertakh, Roshan Weerackcody, Joyce Wong, Ceri Davies, Mark Westwood, Steffen Petersen, Anthony Mathur, Mohiddin Saidi, Neha Sekhri

**Affiliations:** 1 London Chest Hospital, London, UK

## Background

Stress perfusion Cardiac MR is increasingly used in patients with established coronary artery disease (CAD) to guide further management. Our tertiary cardiac centre caters to a population of 1.8 million, performing over 4500 coronary angiograms and 1500 adenosine stress CMR scans/year.

## Purpose

Our aim was to audit our clinical practice to determine the influence of stress CMR imaging on the management of coronary disease and its impact on prognosis.

## Methods

We retrospectively studied 100 consecutive patients with established CAD who underwent adenosine CMR perfusion imaging performed after a median duration of 4 months (IQR: 2.3 - 7months) after their most recent coronary procedure. We examined their hospital electronic records to determine how stress CMR imaging influenced clinical management and outcome.

## Results

Mean age was 62 ± 12 years, and 24% were female. 19 patients had a history of previous coronary artery bypass grafting. A stress perfusion defect in viable myocardium was identified in 71 patients (71%). Of these, 52 (73%) subsequently underwent revascularization by either PCI (n = 49) or CABG (n = 3). Revascularisation was either unsuccessful or not performed in 19 patients (27%) due to the absence of significant symptoms, presence of mild perfusion abnormality or poor revascularisation targets. Among the 29 patients in whom no inducible perfusion defects were identified, revascularization was performed in 3 patients (1%) either due to ongoing symptoms (n = 2) or presence of significant luminal area reduction on intravascular ultrasound (IVUS) (n = 1). Follow-up data was available in 93 patients (93%). During a mean follow-up of 10.2 months, no adverse events were observed in patients without inducible perfusion defects on stress perfusion CMR. Among those patients with inducible perfusion defects on CMR who were treated initially with PCI, one patient died during follow-up and four patients were referred for surgical revascularisation due to significant disease progression/restenosis post PCI.

## Conclusion

Adenosine stress CMR has significantly influenced contemporary management of coronary disease in our centre and forms an invaluable tool to assess ischaemic burden. These findings need to be explored in a larger cohort of patients with longer term follow up.

